# Virtual leadership in relation to employees' mental health, job satisfaction and perceptions of isolation: A scoping review

**DOI:** 10.3389/fpsyg.2022.960955

**Published:** 2022-10-26

**Authors:** Ilona Efimov, Elisabeth Rohwer, Volker Harth, Stefanie Mache

**Affiliations:** Institute for Occupational and Maritime Medicine, University Medical Center Hamburg-Eppendorf, Hamburg, Germany

**Keywords:** leader–employee interaction, virtual leadership, remote work, employee health, virtual collaboration

## Abstract

**Background:**

The significant increase of digital collaboration, driven by the current COVID-19 pandemic, is resulting in changes in working conditions and associated changes in the stress-strain perception of employees. Due to the evident leadership influence on employees' health and well-being in traditional work settings, there is a need to investigate leadership in virtual remote work contexts as well. The objective of this scoping review was to assess the extent and type of evidence concerning virtual leadership in relation to employees' mental health, job satisfaction and perceptions of isolation.

**Method:**

A search was undertaken in five databases, PubMed, Cochrane Library, PsycINFO, PSYNDEX and Web of Science, as well as reference lists of included articles on 9th February 2021 and an update on 28th September 2021. The search strategy was limited to English, German and French language, peer reviewed journal articles published from January 2000 onwards. This scoping review was conducted in accordance with the JBI methodology for scoping reviews. The methodological quality of the included studies was assessed using the JBI critical appraisal tools. A narrative synthesis was conducted.

**Results:**

Nineteen studies met the eligibility criteria for this review. Overarching review findings suggested a positive link between virtual leadership and well-being, job satisfaction, and a negative link to psychological strain, stress and perceptions of isolation of digitally collaborating employees.

**Conclusions:**

By mapping the available evidence on virtual leadership in relation to health and work-related employee outcomes, the review identified many research gaps in terms of content and methodology. Due to limited data, causal relationships were not derived. Future research is needed to examine the complex cause-and-effect relationships of virtual leadership in more detail.

## Introduction

The current global COVID-19 pandemic has had far-reaching impacts on the health and economies of many countries (Chudik et al., 2020; Pfefferbaum and North, 2020). As a result of human mobility restrictions aimed at reducing the incidence of infection, companies have been encouraged to let their employees, mostly white-collar professional workers, work at home if possible (Fadinger and Schymik, 2020; Hernandez and Abigail, 2020). Despite major challenges faced by many companies in adopting digital collaboration from home, a new discussion emerged in society on the distribution of work in the office and at home even for the post-pandemic period (Adams-Prassl et al., 2020; Alipour et al., 2020; Gupta, 2020). Some companies are therefore planning to reduce office space and transform offices into collaborative open workspaces (Alipour et al., 2020) or to redesign the post-pandemic workplace to hybrid ways of working (Fayard et al., 2021; Kane et al., 2021). Yet, the pandemic situation has only intensified an already existing trend. Over the past two decades, a steady increase in the use of digital information and communication technologies (ICTs) and the dissolution of fixed working hours and locations was recorded (Demerouti et al., 2014). From an economic point of view, the rationale for implementing virtual forms of collaboration, namely “New Ways of Working” (NWW) (Demerouti et al., 2014), primarily lies in an increased flexibility and agility of work engagement, an efficient knowledge and information exchange, as well as the reduction of costs, office space, and travel time (Cascio and Shurygailo, 2003; Latniak, 2017; Liao, 2017; Cortellazzo et al., 2019). These benefits can provide a competitive advantage in the globalized market (Hunsaker and Hunsaker, 2008; Kordsmeyer et al., 2019a). On the other hand, these changed working conditions in NWW also pose challenges for virtual collaboration and consequently for leadership. Thus, little or no personal contact due to geographic distance and time differences can lead to social isolation, loss of motivation, difficulties in building trust and team cohesion, and challenges in team coordination or communication (Hunsaker and Hunsaker, 2008; Hertel and Lauer, 2012; Charalampous et al., 2019; Eisenberg et al., 2019; Lengen et al., 2020).

### Virtual leadership

Virtual leadership [or remote leadership or e-leadership (Avolio et al., 2014)] has to adapt to new framework conditions in a digitalized, globalized and highly flexible working environment in which team members interact via digital ICTs and leaders manage their teams across spatial distance, either within time zones or across different time zones (Lilian, 2014). Van Wart et al. (2019) add that leaders should effectively use ICTs by “(1) using ICTs when they are advantageous for various reasons, (2) using the best and most appropriate ICTs available relative to value of various resources, (3) using physically present communication channels when most appropriate, and (4) using ICTs with competence” (Van Wart et al., 2019, p. 83). As such, virtual leadership is not a leadership style, but rather a specific contextual condition for leadership. To date, current research indicated that virtual leadership is more challenging compared to face-to-face leadership due to more difficult relationship building and maintenance as well as coordination of work processes (Akin and Rumpf, 2013; Staar et al., 2019). So far, only little empirical evidence exists on which leadership styles are appropriate for virtual teamwork (Staar et al., 2019), e.g., studies on transformational leadership in virtual teams reveal inconsistent results (Kordsmeyer et al., 2019b). Especially in the course of the COVID-19 pandemic, a mixed form of virtual leadership also increasingly took place: hybrid leadership. Depending on individual needs, some team members are being led completely remotely, while others are being led using a combination of remote and face-to-face leadership (Hopkins and Figaro, 2021). In contrast to virtual leadership, digital leadership describes leadership behavior that drives digital transformation processes in companies (Zeike et al., 2019).

According to current research, leadership in traditional, face-to-face work settings has an evident influence on employee's mental health and job satisfaction (e.g., Kuoppala et al., 2008; Skakon et al., 2010; Montano et al., 2017). In this regard, mental health can be defined as “a state of well-being in which an individual realizes his or her own abilities, can cope with the normal stresses of life, can work productively and is able to make a contribution to his or her community” according to the World Health Organization (WHO) (World Health Organization, 2018, p. 1). This definition involves more than the absence of mental illness [meaning conditions that affect cognition, emotion, and behavior, e.g., depression (Manderscheid et al., 2010)] and constitutes an integral part of a person's health (World Health Organization, 2018). Compared to the significant body of research available on the influence of face-to-face leadership on employees' mental health and job satisfaction, there is still a great need for research on virtual leadership (Avolio et al., 2014; Staar et al., 2019). As virtual collaboration and use of digital ICTs may create psychological stress factors (e.g., acceleration, interruptions or information density) and thereby may have a negative impact on the mental health and job satisfaction of employees (Mache and Harth, 2019), it currently remains unclear which function leaders may have in this context. Reviews conducted hitherto were able to demonstrate for digital, remote collaboration that work environment may play a pivotal role in employees' mental health, job satisfaction, and perceptions of isolation (Charalampous et al., 2019; Oakman et al., 2020). Given the specific contextual conditions of virtual leadership, it seems appropriate to consider also leadership relations to employees' experience of isolation. In the course of COVID-19 related pandemic measures, such as mobility or contact restrictions and consequently a widespread adoption of home office, social isolation emerged as one of the key negative impact factors on mental health (Giorgi et al., 2020; Lengen et al., 2020; Loades et al., 2020) and is significantly negatively related to job satisfaction (Toscano and Zappal, 2020). Social isolation can be defined by “the absence of support from co-workers and supervisors and the lack of opportunities for social and emotional interactions with the team” (Marshall et al., 2007, p. 198). Especially in remote work, limited opportunities for social interaction may also lead to professional isolation, i.e., the reduced possibility of being promoted or rewarded (De Vries et al., 2019). Evidence links between mental health and social isolation also exist apart from pandemic conditions (Leigh-Hunt et al., 2017; Wang et al., 2017) and specifically in geographically dispersed collaboration (Marshall et al., 2007; Wilson et al., 2008).

### Theoretical framework

A recent systematic literature review illustrated that several theoretical concepts and empirical studies on healthy leadership have emerged over the past decade (Rudolph et al., 2020). Thereby, “Health-oriented leadership” (HoL) (Franke et al., 2014) as one dominant concept proved to have a significant impact on employees' mental health (e.g., Kranabetter and Niessen, 2016; Klug et al., 2019; Santa Maria et al., 2019, 2020; Kaluza, 2020; Vonderlin et al., 2021). It represents an integrative and holistic approach that assumes three components influencing the health of the workforce: SelfCare of the leader, StaffCare and SelfCare of the employees. The HoL concept is based on the health-oriented self-leadership of the leader (SelfCare), which consists of three dimensions: value, awareness and behavior. Thus, the leader considers his or her own health to be important, is aware of the stressors in his or her own workplace, and is able to display appropriate health-promoting and preventive behavior. Consequently, the leader's SelfCare influences the health-oriented employee leadership (StaffCare) as well as the employees' SelfCare, all of which also consist of the same three dimensions. According to the HoL model, leaders may influence employees both directly via StaffCare and indirectly in their role model function via their own SelfCare. All three components together (SelfCare of the leader, StaffCare and SelfCare of the employee) enhance the well-being and health of the workforce and consequently reduce perceived stress and health complaints (Franke et al., 2014).

### Study aim

The ongoing need for containment of COVID-19 and continued need to undertake remote work requires evidence synthesis to develop policies and guidelines in order to support virtual leaders in taking care of employees' health. So far, a systematic literature review by Nayani et al. (2018) has outlined the impacts of occupational safety and health leadership on the health of distributed workers. Although it can be assumed that distributed leadership and virtual leadership may have similar associations with the health of employees, little is known about the specific relation between virtual leadership and the mental health, job satisfaction and perceived isolation of geographically dispersed team members with computer workstations. A preliminary search of PubMed, the Cochrane Library and APA PsycINFO was conducted and no current or underway systematic reviews or scoping reviews on the topic were identified. Based on this research gap, this study aims to systematically map the available evidence of virtual leadership in relation to employees' mental health, job satisfaction and perceptions of isolation. Although there are different aspects that can be reviewed with regard to mental health, this systematic review aims to focus on the health-related aspects of mental health as a first step in approaching the subject. Accordingly, due to its scope, performance-related aspects of mental health (e.g., performance, productivity, or engagement) will not be included, as these are distinct areas of research.

Thereby, existing gaps in the literature will be identified and a basis for the development of specific health promotion measurements for digitally collaborating personnel will be created. This scoping review focuses the following research questions:

1. *How is virtual leadership related to employees' mental health, job satisfaction and perceptions of isolation?*
2. *What mediating or moderating variables influence the relation between virtual leadership and employees' mental health, job satisfaction and perceptions of isolation?*

## Materials and methods

### Study design

A scoping review is particularly appropriate for providing an overview of the evidence and covering a broader scope with a variety of study designs. Therefore a scoping review was conducted in accordance with the Joanna Briggs Institute (JBI) methodology for scoping reviews (Peters et al., 2020). The Preferred Reporting Items for Systematic reviews and Meta-Analyses extension for Scoping Reviews (PRISMA-ScR) Checklist was therefore used as reporting guidelines (Supplementary Table 1) (Tricco et al., 2018).

### Search strategy and data sources

Relevant studies were identified through an extensive search on 09th February 2021 in the following five electronic databases: PubMed, Cochrane Library, PsycINFO, PSYNDEX and Web of Science. An update of the literature search was performed on 28th September 2021. Based on the PEO (population, exposure, outcome) scheme, supplemented by criteria for study design and report characteristics (publication type, date, language), a search string with English search words was developed. The search strategy was initially developed for the PubMed database and was later adapted, including all identified keywords and index terms, for each included database and information source (see Supplementary Table 2 for an example full search strategy in one database). Based on the research questions and PEO scheme our search string combined keywords concerning population (e.g., “personnel”, “worker” or “employee”), exposure (e.g., “virtual leadership” or “e-leadership”) and outcome (e.g., “mental health”, “well-being”, “job satisfaction” or “social isolation”). To identify additional relevant studies, searches were further conducted through manual search. Finally, the reference lists of all included sources of evidence were screened for additional studies.

### Eligibility criteria

The study selection was based on predetermined eligibility criteria (Table 1). For inclusion in the present scoping review, studies were required to focus on adult white-collar employees and leaders with computer workstations who regularly collaborate digitally via ICTs during business hours due to spatial difference. Furthermore inclusion-eligible studies had to examine leadership behavior [following a behavioral approach to leadership (Schriesheim and Bird, 1979; Liao, 2017)], and outcomes concerning mental health, job satisfaction or perceptions of isolation. Based on health-related aspects of mental health, outcomes were included that related to absence or presence of mental illness (e.g., depression, burnout) or positive or negative states of well-being (e.g., affective well-being, stress). This scoping review considered qualitative, quantitative, mixed methods, case studies as well as quasi-experimental study research designs for inclusion. Full texts had to be published and made available in languages the research team was capable of (English, German and French). Since the first definition of virtual leadership (“e-leadership”) was published in 2001 (Avolio et al., 2001) and given the need to capture a contemporary work environment, studies published since 2000 were included.

**Table 1:** Eligibility criteria for study selection.

**Criteria**	**Inclusion**	**Exclusion**
Population	Adult white-collar employees and leaders who regularly collaborate digitally via ICTs during business hours due to spatial difference	Self-employed workers, informal working from home or after hours, professionals working in school, medical, or military context
Exposure	Leadership behavior	Selection of media use, linguistic aspects of communication
Outcome	Health-related mental health outcomes, job satisfaction and perceptions of isolation	Physical-related health outcomes, performance, work motivation/engagement, work-life-balance, trust
Study design	Qualitative, quantitative, mixed methods and quasi-experimental study research designs, case studies	Experimental study designs, randomized and nonrandomized controlled trials, reviews
Publication type	Research articles	Reviews, letters, editorials, conference papers, dissertation abstracts, commentaries, reflections, policy statements, books
Publication language	English, German, French	All other languages
Publication date	From 1st January 2000 onwards	Before 1st January 2000

Studies were excluded that focused on self-employed workers, employees who work during regular working hours in the office, but digitally and remotely from home exclusively after hours or during informal working times, or if they were conducted in school, medical, or military contexts as these represent specific work settings in which team structures and leadership roles differ. Studies in which leadership was assessed by selection of media use or linguistic aspects of communication were also excluded, as these studies represent a distinct area of research. Furthermore, studies were excluded that focused on physical-related health outcomes, performance-related aspects of mental health (e.g., performance, productivity, engagement), work motivation, work-life balance or trust as these constructs are distinct to the outcome variables under consideration. Consequently, studies were excluded, if they did not provide an adequate description of the variables under study. Reviews, letters, editorials, conference papers, dissertation abstracts, commentaries, reflections, policy statements, books as well as experimental study designs, randomized and nonrandomized controlled trials were not considered.

### Study selection

Following the search according to the eligibility criteria, all identified citations were collated and uploaded into EndNote 20 (Clarivate Analytics, Boston, USA) and duplicates were removed. Titles and abstracts were then screened by one reviewer (IE) for assessment against the inclusion and exclusion criteria for this review. Full texts of selected citations were assessed in detail by two independent reviewers (IE and ER). Reasons for exclusion of sources of evidence at full text that did not meet the eligibility criteria were documented and reported in the scoping review. Any assessment difficulties or disagreements that arose between the reviewers at each stage of the selection process were discussed in the research team until consent was reached. The interrater reliability was measured by Cohen's kappa statistics using the guideline values according to Altman (1991): κ = < 0.20 poor, κ = 0.21–0.40 fair, κ = 0.41–0.60 moderate, κ = 0.61–0.80 good, κ = 0.81–1.0 very good.

### Data extraction and data analysis

Data was extracted from papers included in the scoping review by one reviewer (IE) and verified by a second reviewer (ER) using a standardized data extraction form developed by the reviewers. The data extracted included general information on authors, year of publication, country, publication type, aims and objectives, study design as well as specific details about the participants, concept, context, exposure, outcomes, data collection instruments and key findings relevant to the review questions. The draft data extraction tool was modified and revised as necessary during the process of extracting data from each included evidence source. The characteristics of the studies were analyzed and summarized in a descriptive manner (**Table 3**). Qualitative data were classified under main categories using narrative synthesis, as suggested by JBI methodology for scoping reviews (Peters et al., 2020) to identify how virtual leadership is related to employees' mental health, job satisfaction and perceptions of isolation. Any disagreements that arose between the reviewers were resolved through discussion in the research team.

### Quality assessment

Conducting a quality assessment is typically not intended for scoping reviews, as these are intended to rather map the evidence (Levac et al., 2010; Rumrill et al., 2010; Munn et al., 2018). Under certain requirements, the assessment of the quality of included studies may also be performed in a scoping review (Peters et al., 2020). In order to identify the risk of diverse biases in included studies and its impact on the validity of inferences, a critical appraisal was performed by two independent reviewers (IE, ER) using JBI standardized appraisal tools (Lockwood et al., 2015; Tufanaru et al., 2020). Based on these checklists, it was assessed whether the quality criteria were (partially) met or not, whether it was unclear or not applicable with justification given for judgement. Any disagreements that arose between the reviewers were resolved by consensus or through discussion in the research team.

## Results

### Included studies and characteristics

**Figure 1:**
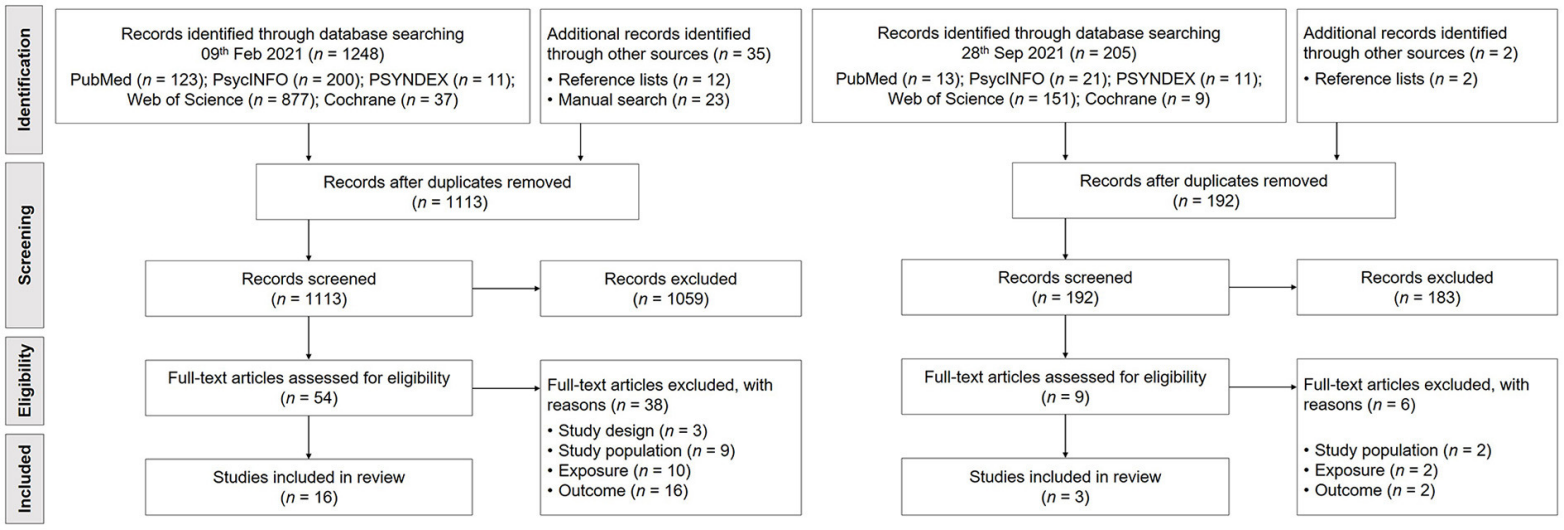
PRISMA-ScR flow diagram of the study selection process.

Overall, 1,248 records were identified through the database search on 09th February 2021 and 35 additional records were derived through manual search and screening of reference lists. After duplicates were removed, 1,113 titles/abstracts were screened by one author (IE). Conclusively, 54 full-text records were assessed by two authors (IE and ER) according to defined eligibility criteria for study selection (Table 1) resulting in 16 included studies. 38 full-text articles were excluded due to unmet criteria for study design (*n* = 3), study population (*n* = 9), exposure (*n* = 10) or outcome (*n* = 16). Cohen's kappa calculated for two raters (IE and ER) on 54 cases yielded κ = 0.61, resulting in a good strength of agreement (Altman, 1991). During the update on 28th September 2021, a total of 192 further records were identified, of which 9 full-text articles were assessed for eligibility by two authors (IE and ER) after screening titles / abstracts (IE). During the analysis of these 9 full-text articles, 6 were excluded due to unmet criteria for study population (*n* = 2), exposure (*n* = 2) or outcome (*n* = 2). Cohen's kappa calculated for two raters (IE and ER) on 9 cases yielded κ = 0.73, resulting again in a good strength of agreement (Altman, 1991). An additional 3 studies were included in the review, resulting in a total of 19 studies. The results of the search and the study selection process are presented in a PRISMA-ScR flow diagram (Tricco et al., 2018) (Figure 1).

Of the 19 included studies, most were conducted in North America (*n* = 9) or Europe (*n* = 7) and were published between 2016 and 2021 (*n* = 9). Almost all studies were cross-sectional (*n* = 18) and conducted as quantitative surveys (*n* = 14). Most studies surveyed teleworkers or telecommuters (*n* = 10) and assessed leadership by means of leadership styles (*n* = 10). Outcome measures were in most studies job satisfaction (*n* = 12) while others examined mental health (*n* = 5) or perceptions of isolation (*n* = 5). Table 2 shows the characteristics of included studies and Table 3 provides an overview of all study characteristics.

**Table 2:** Characteristics of included studies (*n* = 19).

**Characteristic**	**Category**	***n* per category**	**% per category**
Continents	North America Europe Australia / New Zealand South Asia	9 7 2 1	47.4 36.8 10.5 5.3
Year of publication	2000–2005 2006–2010 2011–2015 2016–2021	5 3 2 9	26.3 15.8 10.5 47.4
Study design	Cross-sectional study Longitudinal study	18 1	94.7 5.3
	Quantitative survey study Qualitative interview study Mixed-methods study Quasi-experimental study	14 3 1 1	73.7 15.8 5.3 5.3
Sample	Telework/Telecommuting Virtual teamwork Remote job*	10 5 4	52.6 26.3 21.1
Exposure variables	Leadership styles Leadership support General leadership behaviors or strategies	10 5 4	52.6 26.3 21.1
Outcome variables	Mental health Psychological strain Stress perceptions Well-being Job satisfaction Overall job satisfaction Occupation-specific job satisfaction Perceptions of isolation Social isolation Perceived proximity Professional isolation	5 2 1 2 12 8 4 5 2 1 2	26.3 10.5 5.3 10.5 63.2 42.1 21.1 26.3 10.5 5.3 10.5

*Employees who worked at a different location than their supervisor (e.g., in a different city/state, abroad, or at a customer's site).

**Table 3:** Study characteristics (*n* = 19).

**Reference, country**	**Study design**	**Sample size, population, sex, age**	**Exposure**	**Outcome**	**Main results**	**Critical appraisal score**
Bentley et al. (2016), Australia/New Zealand	Cross-sectional quantitative survey study	804 teleworkers of 28 organizations, 47% female, mean age: 30.9 years	Perceived social support from supervisor	Psychological strain, job satisfaction, social isolation	Negative association between perceived social support (from leaders) and teleworkers' social isolation and psychological strain, positive association with employees' job satisfaction	62.5%
Bregenzer and Jimenez (2021), Austria	Cross-sectional quantitative survey study	1,412 German-speaking employees that face risk factors in digital work (e.g., distributed team work or mobile work), 56.9% female, mean age: 41 years	Health promoting leadership	Employee well-being (perceived stress, work-related resources)	Negative association between health-promoting leadership and employees' stress perceptions, health-promoting leadership moderated the association between risk factors of digital work and stress perceptions of employees	62.5%
De Vries et al. (2019), Netherlands	Longitudinal quantitative diary study	61 teleworkers of a medium-sized Dutch municipality (public servants), 64% female, mean age: 45 years	Daily LMX	Daily professional isolation	High quality LMX moderated the relationship between home-based telework and employees' professional isolation	63.6%
Golden (2006), USA	Cross-sectional quantitative survey study	294 telecommuters of a large telecommunications company, 47% female, mean age: 43 years	LMX quality	Job satisfaction	Statistical partial mediation effects of LMX in the association between extent of telecommuting and employees' job satisfaction	75.0%
Golden and Veiga (2008), USA	Cross-sectional quantitative survey study	375 virtual employees from a large company in the high-tech industry, 45% female, mean age: 42 years	LMX quality	Job satisfaction	Extent of virtual work moderated the positive association between high quality LMX and employees' job satisfaction	62.5%
Haines et al. (2002), Canada	Cross-sectional quantitative survey study	193 telecommuters of 3 companies in the Montreal metropolitan area (federal government agency, a high-tech organization and from one branch of a financial institution), 44% female, mean age: 42.3 years	Supervisor support	Satisfaction with the telecommuting arrangement	Hypothesized positive relationship between supervisor support and satisfaction with telecommuting arrangement not significant	87.5%
Karani and Mehta (2021), India	Cross-sectional quantitative survey study	239 employees working from home during COVID-19 pandemic (consumer durable industry), 40% female, mean age: 35 years	Supervisor support	Emotional and psychological well-being, workspace and life well-being	Indirect positive association between supervisor support and employees' well-being	75.0%
Kelley and Kelloway (2012), Canada	Cross-sectional quantitative survey study	402 employees working in a different city or province/state than manager, 51.3% female, 67% between 30 and 50 years	Transformational leadership	Job satisfaction	Statistical mediation effects of transformational leadership in the positive association between remote work context and employees' job satisfaction.	50.0%
Kirkman et al. (2002), USA	Cross-sectional qualitative interview study	58 team members, 11 team leaders and 6 general managers of 18 virtual teams of a company in the travel industry, sex and mean age NR	Supportive leadership behavior	Isolation/Detachment	Negative association between supportive leadership behavior and employees' feelings of isolation and detachment	70.0%
Konradt et al. (2003), Germany	Cross-sectional quasi-experimental study design	54 full- or part-time teleworkers, 18 non-teleworking employees (control group) with comparable working tasks from the same 19 companies (data processing, telecommunications, financial services), 48,6% female, mean age: 37 years	MBO	Psychological strain, job satisfaction	Negative association between quality of MBO and teleworkers' psychological strain, positive association with their job satisfaction	77.8%
Kurland and Cooper (2002), USA	Cross-sectional qualitative interview study	18 supervisors, 24 telecommuters, 12 non-telecommuters of 2 high-technology firms, 40.7% female, mean age NR	Managerial monitoring strategies	Professional isolation	Negative association between managerial control and telecommuters' professional isolation	70.0%
Kuruzovich et al. (2021), USA	Cross-sectional quantitative survey study	184 teleworkers who telecommuted at least part of the week, sex and mean age NR	LMX	Job satisfaction	Positive association between LMX and teleworkers' job satisfaction, statistical mediation effects of LMX in the association between telecommuting system use and employees' job satisfaction	75.0%
Lurey and Raisinghani (2001), USA	Cross-sectional quantitative survey study	67 employees of 12 virtual teams from 8 companies in the high technology, agriculture, and professional services industries, sex and mean age NR	Internal team leadership	Team members satisfaction	Positive association between internal team leadership and employees' team members satisfaction	37.5%
Madlock (2012), USA	Cross-sectional quantitative survey study	157 non-management telecommuters from 7 companies in the insurance, healthcare, high-tech and banking/finance industry, 48% female, mean age: 31 years	Leadership style	Job satisfaction	Positive association between task-oriented leadership and telecommuters' job satisfaction	75.0%
Mäkelä et al. (2019), Finland	Cross-sectional quantitative survey study	290 Finnish expatriates working in 51 different host countries, 23.3% female, mean age: 42.3 years	LMX (Functional distance)	Satisfaction with expatriate job	Positive association between LMX quality and employees' satisfaction with expatriate job, statistical interaction effects of physical distance to their supervisor in this association	75.0%
Nakrošiene et al. (2019), Lithuania	Cross-sectional quantitative survey study	128 teleworkers exercising different telework intensity, working in IT, insurance, telecommunication sectors, 56% female, mean age: 37.1 years	Supervisor support	Overall satisfaction with telework	Hypothesized positive association between supervisor support and overall satisfaction with telework not significant	62.5%
Poulsen and Ipsen (2017), Denmark	Cross-sectional qualitative interview study	13 distance workers, 4 managers from 4 companies in the software development, engineering or management consultancy, advanced manufacturing, sex and age mean NR	Distance management practices	Employees' wellbeing in distance work	Positive association between leadership behavior and distance workers' well-being	70.0%
Ruiller et al. (2018), France	Cross-sectional qualitative interview study in a mixed methods study design	16 teleworkers, 6 e-leaders from a large company in the telecommunication industry, 40.9% female, mean age NR	E-communicational mode and control management mode (leadership behavior)	Perceived proximity	Positive association between e-communicational mode of leaders (described as high leader-member-exchange behavior) and teleworkers' sense of belonging	60.0%
Whitford and Moss (2009), Australia	Cross-sectional quantitative survey study	165 employees from a broad, random sample of small, medium, and large public and private organizations, 47% working at a different location than supervisor, 45% female, 53% between 25 and 34 years	Transformational leadership	Job satisfaction	Positive association between visionary leadership and employees' job satisfaction, employees' promotion focus and spatial distance moderated the association between visionary leadership and employees' job satisfaction	87.5%

NR, not reported; MBO, Quality of Management by Objectives; LMX, Leader-Member-Exchange.

### Quality assessment

According to Altman (1991), the inter-rater reliability based on Cohen's kappa (κ = 0.74 for the initial search, κ = 0.89 for the update) indicated a good to very good strength of agreement. Almost all analytical cross-sectional studies (*n* = 12) achieved at least 50.0% of the criteria, with only one study fulfilling 3 out of 8 quality criteria (37.5%) of the Critical Appraisal checklist. All four included qualitative studies met at least 6 out of 10 quality criteria (60.0%) of the checklist. The cohort study of this review fulfilled 7 out of 11 quality criteria (63.6%) and the quasi-experimental study fulfilled another 7 out of 9 quality criteria (77.8%) of the checklist. Following the objective of a scoping review, the quality assessment of these studies did not result in any exclusion from the review (Peters et al., 2020). Quality assessment results for all included studies are presented in Supplementary Tables 3–6.

### Synthesized findings

#### Virtual leadership in relation to mental health outcomes of employees

Two studies showed positive links between general (Poulsen and Ipsen, 2017) or supportive leadership behaviors (Karani and Mehta, 2021) and employee well-being. Karani and Mehta (2021) displayed that supervisor support of employees who worked from home during the COVID-19 pandemic was positively associated with employees' well-being in an indirect way in that the supervisor support was positively linked to employees' psychological contract fulfillment (β = 0.12, *p* < 0.05), psychological contract fulfillment was positively linked to work engagement (β = 0.77, *p* < 0.05), and work engagement was positively linked to psychological (β = 0.66, *p* < 0.05) and emotional well-being (β = 0.57, *p* < 0.05) (Karani and Mehta, 2021). Yet again, a qualitative interview study by Poulsen and Ipsen (2017) revealed that inter-organizational distance workers (work at customers' sites) perceived both positive and negative states of well-being. Thus, on the one hand, they experienced flexibility, autonomy, and potential for personal growth in their work; on the other hand, poor physical and social working conditions were reported to be linked to perceptions of social isolation, loneliness, frustration, and unmet basic needs (e.g., physiological needs and safety). A qualitative analysis of leadership behaviors revealed that dialogue, feedback, and strong managerial perceptual skills were experienced as beneficial in relation to positive well-being (Poulsen and Ipsen, 2017).

Furthermore, two other studies investigated negative associations between psychological strain and quality of management by objectives (MBO) (Konradt et al., 2003) or supportive leadership behaviors (Bentley et al., 2016). Therefore, the results of Konradt et al. (2003) indicated in their cross-sectional study that MBO, a delegative management concept, had the highest statistical predictive value [independent of other (non-) job-related stressors] for perceived psychological strain of teleworkers (ß = −0.42, *p* < 0.01). According to this, employees' irritation was negatively associated with perceived quality of their leadership (MBO), with high quality of MBO representing clearer goals, more feedback, and a higher degree of participation (Konradt et al., 2003). Bentley et al. (2016) demonstrated that perceived social support (from leaders) was negatively associated with psychological strain of teleworkers (ß = −0.28, *p* < 0.001). In addition, statistical mediation effects in the cross-section on social isolation due to teleworking at home were demonstrated in this association (Bentley et al., 2016).

And lastly, the study by Bregenzer and Jimenez (2021) revealed that risk factors of digital work (distributed team work, mobile work, constant availability, and inefficient technical support) were positively associated with stress perceptions among employees. Health-promoting leadership was negatively associated with stress perceptions (ß = −0.31, *p* < 0.001). Additionally, statistical moderation effects on health-promoting leadership in the association between stress perceptions and two risk factors (mobile work^*^health-promoting leadership: ß = 0.11, p < 0.001; inefficient technical support^*^health-promoting leadership: ß = 0.07, p < 0.03) were reported (Bregenzer and Jimenez, 2021).

#### Virtual leadership in relation to job satisfaction of employees

The studies on overall job satisfaction almost exclusively examined positive associations to established leadership styles, namely Leader-Member-Exchange (LMX) (Golden, 2006; Golden and Veiga, 2008; Kuruzovich et al., 2021), task-oriented leadership (Madlock, 2012), transformational leadership (Whitford and Moss, 2009; Kelley and Kelloway, 2012), and MBO (Konradt et al., 2003). Another study on overall job satisfaction examined a positive association to supportive leadership behaviors (Bentley et al., 2016). Following studies on the link between relationship-oriented leadership styles and overall job satisfaction exhibited contrasting results: A cross-sectional survey study by Golden (2006) showed statistical partial mediation effects for relationship quality with the leader (measured by LMX) in the association between extent of telecommuting and employees' job satisfaction (ß = −0.16, *p* < 0.01; Δ*R*^2^ = 0.02, *p* < 0.001). Furthermore, Golden and Veiga's cross-sectional survey study found statistical moderation effects for extent of virtual work in the positive association between LMX and job satisfaction (ß = 0.20, *p* < 0.001) (Golden and Veiga, 2008). Likewise, more recent study results of Kuruzovich et al. (2021) showed a positive link between LMX and job satisfaction (ß = 0.24, *p* < 0.01) and statistical mediation results in the cross-section for LMX in the association between telecommuting system use and employees' job satisfaction. Furthermore, statistical moderation results were reported for telecommuting software quality in this association. Contrasting results were shown by Madlock (2012) in his cross-sectional survey study: relationship-oriented leadership was reported to have no statistical predictive value, but task-oriented leadership to have the highest statistical predictive value for job satisfaction among telecommuters (ß = 0.57, *p* < 0.001). Studies on transformational leadership, a motivational and visionary leadership style, also suggested that this leadership style was positively associated with employees' overall job satisfaction in remote work contexts: Kelley and Kelloway (2012) cross-sectional survey study illustrated statistical mediation effects of transformational leadership style in the positive association between remote work context and employees' job satisfaction. Similarly, Whitford and Moss (2009) cross-sectional survey study of employees working in distributed teams indicated that visionary leadership (subscale of transformational leadership) was positively associated with job satisfaction when employees' promotion focus was sufficiently high. Statistical moderation effects for promotion focus as well as spatial distance were reported for the positive association between visionary leadership and job satisfaction (Whitford and Moss, 2009). Moreover, quasi-experimental study results of Konradt et al. (2003) showed that MBO as a delegating leadership style had the highest statistical predictive value for job satisfaction among teleworkers (ß = 0.52, *p* < 0.01). In comparison to studies on leadership styles, Bentley et al. (2016) quantitative survey indicated that perceived social support (from leaders) was also positively associated with job satisfaction (ß = 0.40, *p* < 0.001).

Additional four studies examined the positive link between LMX leadership style (Mäkelä et al., 2019), internal team leadership (Lurey and Raisinghani, 2001) or supervisor support (Haines et al., 2002; Nakrošiene et al., 2019) and employees' occupation-specific job satisfaction. The scales used assessed satisfaction with the specific occupation (e.g., telecommuter or expatriate). Similar to study results on overall job satisfaction, the cross-sectional survey study conducted by Mäkelä et al. (2019) showed a positive association between LMX quality and job satisfaction with expatriate job (ß = 0.22, *p* < 0.05). Furthermore, statistical interaction effects in the cross-section were reported between LMX quality and physical distance (ß = 0.22, *p* = 0.05), indicating positive associations between LMX quality and job satisfaction with expatriate job when physical distance to supervisor was low (e.g., by still working in the same time zone or culture despite being sent abroad as an expatriate) (Mäkelä et al., 2019). Another study by Lurey and Raisinghani (2001) illustrated that internal team leadership and team members' satisfaction was positively associated (*r* = 0.45, *p* < 0.01). In contrast, two studies by Haines et al. (2002) and Nakrošiene et al. (2019) found no significant associations between supervisor support and satisfaction with telecommuting arrangement (Haines et al., 2002) or telework (Nakrošiene et al., 2019).

#### Virtual leadership in relation to perceptions of isolation of employees

Two studies found positive associations between spatial distance in teams and perceptions of professional isolation among employees (Kurland and Cooper, 2002; De Vries et al., 2019) (i.e., reduced possibility of being promoted or rewarded at the workplace). De Vries et al. (2019) conducted a longitudinal quantitative diary study and demonstrated that home-based teleworking resulted in higher levels of professional isolation among public servants and that a high-quality LMX was conducive in reducing this effect. In addition, an interview study with telecommuters from the high-tech industry by Kurland and Cooper (2002) revealed that regular telecommuting was reported to be positively associated with perceptions of professional isolation among employees. The results of qualitative triad analyses indicated that managerial control was linked to employees' professional isolation. Accordingly, study participants' long-term professional development was described to suffer if they were not given regular opportunities to present ideas, to network, or if leaders faced difficulties with remote mentoring or focused only on results in the short term (Kurland and Cooper, 2002).

Furthermore, two other studies evaluated the negative link between supportive leadership behaviors and perceptions of social isolation (Kirkman et al., 2002; Bentley et al., 2016). A cross-sectional survey study by Bentley et al. (2016) indicated that perceived social support (by leaders) was negatively associated with perceptions of social isolation (ß = −0.46, *p* < 0.001). This association was statistically reported to be greater for low-intensity teleworkers (< 8 h telework/week; ß = −0.39, *p* < 0.001) than for higher-intensity teleworkers (> 8 hours telework/week; ß = −0.60, *p* < 0.001) (Bentley et al., 2016). Additionally, a qualitative interview study with virtual team members, leaders, and general managers at a U.S. company by Kirkman et al. (2002) elaborated that virtual leaders counteracted team members' feelings of isolation and detachment by proactively and frequently communicating with them (e.g., routine phone calls or e-mails), by building mentor-protégé relationships, and facilitating face-to-face meetings (e.g., via teambuilding or company events, increased client contact by redesigning job assignments, or by encouraging networking within the company). Especially at the beginning of virtual teamwork, leaders acknowledged that minimal communication was misunderstood as a positive sign and that it took time to realize how to deal with employees' social isolation (Kirkman et al., 2002).

Additionally, interview results with teleworkers and their leaders by Ruiller et al. (2018) illustrated that leadership behavior was linked to perceived proximity in distributed teams and that leaders were experienced to have a responsibility in preventing de-proximity risks. According to these findings, leadership was reported to be positively related to team members' sense of belonging as leaders worked as part of the team in an equally distributed manner and thus shared the same experiences, jointly developed team goals and structures, build a team identity, and established high quality face-to-face and distant communication (Ruiller et al., 2018).

## Discussion

The aim of this scoping review was to systematically map the available evidence of virtual leadership in relation to employees' mental health, job satisfaction and perceptions of isolation and thereby identify existing gaps in the literature. Referring back to the research questions concerning the relations between virtual leadership and employees' mental health, job satisfaction and perceptions of isolation as well as mediating and moderating variables in this relation, overarching review findings suggested a positive link between virtual leadership and mental health, job satisfaction, and a negative link to perceptions of isolation of digitally collaborating employees. Virtual leadership was found to be both directly and indirectly related to employees' mental health, job satisfaction, and perceptions of isolation. To date, the cause-and-effect relationships of virtual leadership are very complex and still remain unclear, especially in relation to employees' mental health and perceptions of isolation. Reflecting upon our theoretical framework, the HoL model (Franke et al., 2014), it can be noted that all of the included studies only examined follower-directed virtual leadership (i.e. “StaffCare”) in relation to employee outcomes. So far, no studies have been conducted that holistically examined self- and follower-directed leadership (i.e. “SelfCare leader”, “SelfCare followers” and “StaffCare”).

### Virtual leadership in relation to mental health outcomes of employees

With regard to mental health, five studies indicated that leaders using a high quality MBO, a health-oriented leadership style, or supportive leadership behaviors were negatively associated with psychological strain (Konradt et al., 2003; Bentley et al., 2016) and stress perceptions (Bregenzer and Jimenez, 2021) and were positively associated with well-being of employees (Poulsen and Ipsen, 2017; Karani and Mehta, 2021). The associations of virtual leadership and mental health outcomes examined in these studies suggested social isolation (Bentley et al., 2016) and work engagement (Karani and Mehta, 2021) as potential mediators. According to present review results, there is no evidence on the relation between virtual leadership and mental illnesses. Moreover, it can be further discussed whether virtual leadership may act as a buffer by mitigating the negative relation between digital work and employees' stress perception (see Bregenzer and Jimenez, 2021). More recent findings that were unable to meet our eligibility criteria due to sample composition still yielded interesting aspects for our review. Dolce et al. (2020) study of virtual leadership in relation to mental health showed that destructive leaders in ad hoc telework during the pandemic were positively associated with employees' cognitive demands, a pressing use of technology, a reduction in autonomy, and, consequently, exhaustion and impaired recovery. Similarly, a recent study revealed that intrusive leadership was positively associated with the stress of workaholic employees who telecommuted after hours, and was also linked to decreased happiness, anxiety and depression (Magnavita et al., 2021). A recent systematic review highlighted the risk of negative psychological impacts of NWW on employees (e.g., blurred work-home boundary or fatigue) (Kotera and Correa Vione, 2020), which increases the overall relevance of promoting health protective factors for digitally collaborating employees. Despite limited number of studies in the present review, some similar results to the current state of research on face-to-face leadership were found. Several systematic reviews and meta-analyses demonstrated that face-to-face leadership can act as both a resource and a stressor (Gregersen et al., 2011) and can have a decisive influence on employees mental health (Kuoppala et al., 2008; Skakon et al., 2010; Montano et al., 2017) and leaders themselves (Kaluza et al., 2020). Most notably, these reviews reported transformational or relationship-oriented leadership styles to be positively related to mental health outcomes of employees (Gregersen et al., 2011; Montano et al., 2017).

### Virtual leadership in relation to job satisfaction of employees

Furthermore, with regard to job satisfaction, present review findings indicated a positive association between virtual leadership [as surveyed by LMX quality (Golden, 2006; Golden and Veiga, 2008; Mäkelä et al., 2019; Kuruzovich et al., 2021), task-oriented leadership (Madlock, 2012), transformational leadership (Whitford and Moss, 2009; Kelley and Kelloway, 2012), quality of MBO (Konradt et al., 2003)], supportive [(Bentley et al., 2016) or general leadership behaviors (Lurey and Raisinghani, 2001)] and employees' job satisfaction. Although most identified studies in this review examined employee job satisfaction as an outcome [see Charalampous et al. (2019) for similar results], the direction of this potential link is not clear. Namely, two studies found no significant association (Haines et al., 2002; Nakrošiene et al., 2019). Contrasting results were found between the studies on the positive link between LMX and job satisfaction (Golden, 2006; Golden and Veiga, 2008; Mäkelä et al., 2019; Kuruzovich et al., 2021) and a study that found no significant association between relationship-oriented leadership and job satisfaction (Madlock, 2012). Moreover, it is not clear how virtual leadership may affect employees' job satisfaction, as most studies assumed a direct link to job satisfaction but others indicated virtual leadership to serve as a mediator (Golden, 2006; Kelley and Kelloway, 2012; Kuruzovich et al., 2021) or moderator (Golden and Veiga, 2008) in the association between remote context variables and job satisfaction. In addition, two studies reported spatial distance, employees' promotion focus (i.e. orientation toward potential benefits or gains, rather than potential losses) and extent of digital work to be potential moderators influencing the link between virtual leadership and employees' job satisfaction (Whitford and Moss, 2009; Mäkelä et al., 2019). The relation between virtual leadership and employees' job satisfaction could therefore be dependent on other contextual factors. Yet, there are diverging research results on the impact of remote working conditions on employees' job satisfaction (Golden and Veiga, 2005). For example, it can be discussed whether telecommuting software quality can contribute to an improved telecommuting experience (Kuruzovich et al., 2021). Previous research more specifically discussed a curvilinear relationship between the extent of digital, remote work and job satisfaction (Charalampous et al., 2019). Outside our scope due to sample characteristics, a study of early-career professionals recruited from a Masters of Business Administration program suggested that the extent of electronic communication at work enhanced the positive association between LMX and students' job satisfaction (Hill et al., 2014). Thus, virtual work characteristics may influence the associations between leadership and employee outcomes. While the current state of research supports a significant direct influence of face-to-face leadership on job satisfaction (Kuoppala et al., 2008; Skakon et al., 2010), the present results can only point to a potential link between virtual leadership and employees' job satisfaction, since only quantitative cross-sectional studies have been conducted so far. To some extent, the comparability of these study results remains questionable, as differing (extensive) measurement instruments were used and recent rapid technological developments over the last two decades have changed the context conditions, in some cases considerably. For example, in the study by Lurey and Raisinghani (2001), study participants stated that they had hardly used videoconferencing. Accordingly, it can be concluded from the comparison of the included studies that the technical requirements and possibilities of virtual leadership differed greatly.

### Virtual leadership in relation to perceptions of isolation of employees

Lastly, regarding review results on perceptions of isolation, preliminary review results suggested that employees in remote workplaces are more likely to experience isolation (Kurland and Cooper, 2002; De Vries et al., 2019) – as well as leadership behavior may reduce this perception of social and professional isolation of digitally collaborating employees (Kirkman et al., 2002; Kurland and Cooper, 2002; Bentley et al., 2016; Ruiller et al., 2018; De Vries et al., 2019). However, present review results indicated that supportive leadership behavior from a distance may also be experienced as challenging by leaders (Kirkman et al., 2002; Kurland and Cooper, 2002; Ruiller et al., 2018). The available studies did not indicate any moderating or mediating variables in this association. Similar findings, except our scope due to insufficiently defined sample composition, emerged from another two studies with transformational or considerate leadership reporting negative associations with sales peoples' perceptions of isolation (Mulki and Jaramillo, 2011; Munir et al., 2016). Moreover, a study among distributed workers found that workplace inclusion strengthened the positive relation between health-and-safety-specific leadership and self-rated health (Nielsen et al., 2019). The study findings of Golden et al. (2008) also illustrated that teleworkers' working conditions were related to perceptions of isolation. The negative association between teleworkers' professional isolation and their job performance was enhanced by extent of teleworking, whilst face-to-face interactions and access to communication-enhancing technology reduced this effect. A recent review showed that workplace isolation in digital, remote work settings is related to various employee outcomes, including emotional exhaustion, well-being and job satisfaction (Sahai et al., 2020). Sahai et al. (2020) argued in their review that the constructs of social and professional isolation are despite their differences intertwined and therefore combined them in the term workplace isolation.

### Overarching discussion of review results

Due to the fact that no causal relationships of virtual leadership can yet be derived, it remains unclear how the influence of various leadership styles on health and work-related employee outcomes differs in virtual collaboration. However, what is apparent from the results is that context is decisive for virtual leadership and requires an adaptation of leadership behavior. In this regard, the working conditions of NWW can vary greatly, e.g., in terms of technical requirements and equipment, extent of working virtually and remote, organizational support or corporate culture (Kirkman et al., 2002; Bentley et al., 2016; Nakrošiene et al., 2019; Kotera and Correa Vione, 2020; Niebuhr et al., 2022). Thereby, the COVID-19 pandemic poses a special context for virtual, remote collaboration. It has increased employees' experiences of isolation and related negative mental health consequences in ad hoc remote work, for example with negative effects on stress experience, experienced remote work productivity and remote work satisfaction, but also on social relationships at work (Toscano and Zappal, 2020; Carillo et al., 2021; Galanti et al., 2021; Xiao et al., 2021). Of all included studies, only one examined leadership in relation to employee well-being in remote work due to the COVID-19 pandemic (Karani and Mehta, 2021). Another recent interview study by Yarberry and Sims (2021) explored, amongst others, educators' experiences with prompted virtual teamwork during the COVID-19 pandemic. Again, employees reported feelings of depression due to social isolation, lack of human connection or belongingness. Likewise, leaders' support was attributed a key role by demonstrating their care and concern and finding a balance between micro-management and excessive attention (Yarberry and Sims, 2021). Previous studies indicated that the contextual conditions for virtual leaders differed greatly before and during the pandemic (Chamakiotis et al., 2021). Leaders were often challenged to adjust to ad hoc remote leadership (as well as increasingly hybrid forms of work) without prior knowledge (Chamakiotis et al., 2021) and were confronted with increased stress experiences of employees due to the pandemic situation (Evanoff et al., 2020). Additionally, characteristic of virtual teams in the pandemic is that employees often had to switch from traditional face-to-face to virtual collaboration unprepared in both technical (e.g., lack of organizational readiness) and mental terms (e.g., work-family conflicts) (Chamakiotis et al., 2021).

### Implications for future research

In general, the review highlights that there are insufficient research findings to draw generalizable conclusions on virtual leadership in relation to health-related aspects of mental health, job satisfaction and perceptions of isolation of employees. As a result of this systematic review, by capturing key findings, broader clusters for future research could be identified and, consequently, recommendations for future research could be mapped. As there is still a great need for research identified in this review, it is recommended for future research to examine which leadership behaviors have the strongest impact on health-related outcomes. In this context, it will be crucial for future research to investigate whether it remains necessary to adapt existing leadership styles to the specific contextual conditions of virtual, remote work or whether a completely new leadership approach is required. Moreover, it should be investigated whether the effect of different leadership styles differs in the context of virtual, remote collaboration (e.g., does it take behaviors of different leadership styles, e.g., both, relationship-oriented and transformational leadership? Or is a specific leadership approach related to health needed, e.g., HoL by Franke et al., 2014?). Especially in the context of virtual leadership in pandemic conditions, it is important to explore whether the conditions and thus leadership behaviors differ (e.g., dealing with alternating virtual and hybrid leadership).

Furthermore, future empirical studies should investigate the impact of virtual leadership on various employee outcomes in relation to mental health (e.g., stress and strain perception, well-being, mental illnesses, but also performance-related aspects of mental health), job satisfaction and perceptions of isolation (e.g., social and professional isolation) using validated instruments (e.g., Cammann et al., 1979; Maslach and Jackson, 1981; Golden et al., 2008). Following the review by Sahai et al. (2020), future studies could also examine workplace isolation as a multi-dimensional construct, assessing both social and professional isolation (Marshall et al., 2007). Given the potential link between the experience of isolation and mental health identified in this review, future research should examine both constructs in relation to virtual leadership (Bentley et al., 2016).

Referring to the theoretical HoL model used in this review (Franke et al., 2014), it is recommended to examine leadership influence in virtual collaboration more holistically: both directly via follower-directed leadership (i.e., StaffCare) and indirectly via self-directed leadership (i.e., SelfCare from both leaders and employees). The exploration of employees' SelfCare is an interesting aspect of future research, especially in the context of virtual collaboration [e.g., in dealing with various stressors, including techno-stressors (Rohwer et al., 2022)], as well as in light of the fact that employees are active participants in a reciprocal leader-follower relationship (Mäkelä et al., 2019). In addition, virtual collaboration is often organized in a decentralized way, changing the role of the leader and making it more difficult for leaders to directly influence all of their followers' stressors. Furthermore, considering increased health risks of leaders (e.g., greater exhaustion or higher professional or quantitative overload) (Pangert and Schüpbach, 2011; Lohmann-Haislah, 2012; Stilijanow, 2012) and a significant association between leaders' health status and leadership behavior (Kaluza et al., 2020), it is recommended to investigate health-oriented self-leadership among leaders as well. Future studies should examine the impact of virtual leadership on leaders' health outcomes (see Efimov et al., 2020) as well as interdependencies between employees' and leaders' health status in virtual collaboration [e.g., by using health-oriented leadership measurement instrument by Franke et al. (2014)]. In this regard, future interventional research or experimental studies should investigate the relationship between health-oriented leadership and e-health literacy skills: What skills do leaders need in order to discuss health-related topics in virtual communication or to reduce employees' perceived isolation? In general, future research should further focus on cause-effect relationships of virtual leadership and investigate under which conditions and characteristics virtual leadership functions as a resource or stressor.

The studies presented in this review illustrated that the impact of virtual leadership may also depend on different contributing factors (e.g., individual, organizational, social or technical factors). For this purpose, studies on virtual leadership should also examine different working conditions in companies (e.g., with respect to technical equipment, corporate culture, organizational support or social support by coworkers). Thereby, further research should examine the impact of the extent of remote digital work on employees' perceptions of virtual leadership. As the COVID-19 pandemic has strongly driven the digitalization of workplaces, employees' experiences may differ compared to before the pandemic (Niebuhr et al., 2022). Similarly, studies should be conducted in different sectors and countries to identify potential differences. Accordingly, studies should investigate whether the effect of virtual leadership may function more strongly as a buffer for employees in industries with increased job demands. Given that most studies in this review were conducted in North American or European countries, future studies should also be conducted in diverse countries and cultures. Furthermore, on an individual level, employees' individual needs and characteristics should be taken into account in relation to virtual leadership perception and employee outcomes. Considering diverse factors, future studies should analyze which predictors have the greatest impact on employees' health- and work-related outcomes.

Overarching recommendations include different study designs based on qualitative and quantitative methods or mixed-methods designs. Due to the fact that most of the included studies were quantitative, future qualitative research is needed to explore potential influencing factors and relationships to virtual leadership and health-related outcomes. Given that the majority of studies in this review were cross-sectional, there is a great need for longitudinal and intervention studies in order to obtain generalizable results and to identify causalities in the cause-effect relationships of virtual leadership, as well as to be able to interpret mediation effects.

### Implications for practice

At an organizational level, organizations in general are recommended to holistically design, carefully and participatory plan, continuously evaluate, adapt and adjust the introduction of NWW (Kirkman et al., 2002; De Vries et al., 2019). Thereby, organizations should recognize that leadership can also be a relevant influencing factor for employees' mental health, their job satisfaction and perceptions of isolation in virtual, remote collaboration. Basically, organizations should be aware that no leadership or merely transferring traditional leadership in virtual, remote collaboration can pose risks (Contreras et al., 2020). Preparing leaders for health-promoting and effective leadership in virtual, remote collaboration requires organizational support at different levels. In this regard, it should be taken into account that more organizational support for leaders and employees is needed in teams with a higher degree of virtual, remote collaboration (Haines et al., 2002; Kurland and Cooper, 2002; Golden, 2006; Bregenzer and Jimenez, 2021). In general, the review results indicate that behavioral interventions for virtual leadership require adaptation to the specific contextual conditions of virtual, remote collaboration. Although research at this stage does not allow evidence-based recommendations on the application of specific leadership styles, the present review results indicate that certain behavioral activities should be promoted. It is recommended that virtual leaders develop and maintain high quality relationships to their dispersed team members by learning socio-emotional and communicative skills in order to build trust and respect in their team, to establish a shared identity, to enable participation, and to communicate effectively and sensitively via ICTs (Golden and Veiga, 2008; Ruiller et al., 2018; De Vries et al., 2019; Mäkelä et al., 2019). This requires leaders to learn how to communicate effectively via digital ICTs and to select the appropriate communication media accordingly. In doing so, it is crucial to engage in both planned and unplanned communication, as well as in formal and informal communication (Kelley and Kelloway, 2012; Ruiller et al., 2018). Organizations are required to offer a good quality and quantity of technical equipment to the workforce to enable effective communication. Social technologies should also be considered for social exchange (Kuruzovich et al., 2021). Adapting leadership behavior to the virtual, remote context takes work and time, as it takes experience for leaders to correctly interpret the different and individual communication cues to recognize, e.g., perceptions of isolation, stress or strain among employees (Kirkman et al., 2002; Ruiller et al., 2018). In view of the fact that leaders in virtual teamwork are attributed a more moderating role (Hertel and Orlikowski, 2018; Staar et al., 2019), it is also recommended by the present review results that leaders apply a leadership style that uses delegation principles (Konradt et al., 2003). Thereby, they can promote task autonomy and contribute to employee well-being (Mäkelä et al., 2019). In addition, the relevance of clear communication of goals, tasks and responsibilities is increasing, especially against the backdrop of various communication difficulties in virtual collaboration (Lurey and Raisinghani, 2001; Madlock, 2012). In order to achieve long-term behavioral changes in organizations, both behavioral interventions and structural measures need to be implemented. On the one hand, virtual teams should be given the opportunity to hold face-to-face meetings at the beginning of a collaboration or at regular intervals (Kelley and Kelloway, 2012; Mäkelä et al., 2019), and to participate in company-wide networks on best practices (e.g., also platforms for informal exchange) (Kirkman et al., 2002; Kurland and Cooper, 2002). On the other hand, preventive occupational health and safety programs should be offered to employees and leaders in organizations, along with the development of a universal communication culture and etiquette for digital collaboration. Moreover, corporate structures should be adapted, hierarchies reduced and an open and agile corporate culture developed in order to establish an appropriate working environment in which virtual health-promoting leadership may have an impact (Contreras et al., 2020).

At an individual level leaders should be aware of the responsibility and influence they have on employees. Especially in digital communication, leaders should learn to be even more sensitive to the emotions and different needs of their team members and to respond accordingly (Kirkman et al., 2002; Bregenzer and Jimenez, 2021). Of equal importance is that virtual leaders develop an awareness of the challenges associated with the specific working conditions (e.g., time differences, language barriers, communication problems, lack of trust) (Poulsen and Ipsen, 2017; Mäkelä et al., 2019). Overall, it should be noted that successful change processes at the individual or even organizational level take time to develop.

### Strengths and limitations

To the best of our knowledge, this is the first review which aims to systematically map the available evidence of virtual leadership in relation to employees' mental health, job satisfaction and perceptions of isolation. In preparing this review, the JBI methodology for scoping reviews (Peters et al., 2020) and the PRISMA-ScR Checklist (Tricco et al., 2018) were closely followed. Accordingly, the study selection was conducted systematically and conscientiously by two independent researchers, which improved the reliability of the findings. The selection of narrow inclusion criteria ensured that only studies that actually investigated virtual, remote leadership (e.g., in contrast to distributed or digital leadership) were included. For the purpose of a scoping review, the aim of this review was to map the evidence (Levac et al., 2010; Rumrill et al., 2010; Munn et al., 2018). Accordingly, this review was able to systematically provide an overview of the field and, in particular, to derive recommendations for future research based on the content and methodological analysis of the included studies. This resulted in the identification of broader clusters for future research. Considering the still emerging research field, the review offers valuable approaches for future research directions.

Among the limitations of this review, it must be noted that, first, to date there is limited evidence on virtual, remote leadership in relation to employees' mental health, job satisfaction and perceptions of isolation and, second, the available data remains heterogeneous despite narrow inclusion criteria. It remains questionable whether the conditions of virtual, remote work were comparable for all included samples. For example, differences are possible between teleworkers, telecommuters, expatriates, or virtual team members (e.g., does only one team member or do all team members work remotely?), as these terms are often used interchangeably (Bailey and Kurland, 2002; Nakrošiene et al., 2019). There may also be differences in individual working conditions, such as different levels of telework intensity (e.g., full-time or part-time), the remote work location (e.g., home office, customer site or abroad) and different technical equipment. Moreover, it remains unknown whether differences in collaboration existed across national or cultural boundaries. There are also differences in the methodological quality of the included studies. The instruments used to survey outcomes in the included studies vary in quality (e.g., self-developed or validated items). Similarly, it was found that diverse instruments of varying quality were applied to assess leadership in virtual, remote settings (general leadership behaviors, social support by leaders, or established leadership styles), but none of them used instruments specifically adapted to virtual leadership. In some cases, access to the survey items was not provided, ethical considerations were not always listed, or the research was financed by a company. Lastly, despite limited interpretation (O'Laughlin et al., 2018), mediation analysis of cross-sectional data were reported and discussed in the present review.

## Conclusions

Given the increased trend toward virtual, remote work due to the COVID-19 pandemic, the aim of this scoping review was to examine the current state of research on virtual leadership in relation to employees' mental health, job satisfaction and perceptions of isolation. A total of nineteen studies were identified, which indicated a positive link between virtual leadership and well-being, job satisfaction, and a negative link to psychological strain, stress and perceptions of isolation of digitally collaborating employees. Due to limited data, causal relationships were not derived. By mapping the current state of research, the review was able to identify numerous research gaps in terms of content and methodology that need to be addressed in the future. Future research is needed to examine the complex cause-and-effect relationships of virtual leadership and its impact on health-related outcomes of employees in more detail. Recommendations for practice in promoting healthy virtual leadership in organizations include supporting leaders via behavioral und structural interventions in order to raise awareness for their responsibilities and impact as well as to enable leaders to implement leadership behavior adapted to the specific contextual conditions of virtual, remote collaboration.

## Data availability statement

The original contributions presented in the study are included in the article/Supplementary material, further inquiries can be directed to the corresponding author.

## Author contributions

Conceptualization, writing—review and editing, and project administration: IE, ER, VH, and SM. Methodology and full-text screening: IE and ER. Title and abstract screening, writing—original draft preparation, and visualization: IE. Supervision: VH and SM. All authors have read and agreed to the published version of the manuscript.

## Conflict of interest

The authors declare that the research was conducted in the absence of any commercial or financial relationships that could be construed as a potential conflict of interest.

## Publisher's note

All claims expressed in this article are solely those of the authors and do not necessarily represent those of their affiliated organizations, or those of the publisher, the editors and the reviewers. Any product that may be evaluated in this article, or claim that may be made by its manufacturer, is not guaranteed or endorsed by the publisher.

## Supplementary material

Supplementary Table 1 PRISMA-ScR Checklist.

Supplementary Table 2 Search strategy for PubMed.

Supplementary Tables 3–6 Critical appraisal results.
