# Supplementary material for: Virtual leadership in relation to employees' mental health, job satisfaction and perceptions of isolation: A scoping review
Source: Front Psychol. 2022 Oct 26;13:960955. doi: 10.3389/fpsyg.2022.960955 (PMC9644018; doi:10.3389/fpsyg.2022.960955)
Supplement: Supplementary Table 2 — Search strategy for PubMed. [file Table_2.pdf]

Supplementary Table 2. Search strategy for PubMed (U.S. National Institutes of Health's National Library of Medicine (NIH/NLM)).

#1	"personnel"[Text Word] OR "worker*"[Text Word] OR "staff"[Text Word] OR "employee*"[Text Word] OR "professional*"[Text Word] OR "job"[Text Word] OR "work*"[Text Word] OR "employment"[Text Word] OR "occupation*"[Text Word]
#2	"leader*"[Title/Abstract] OR "supervisor*"[Title/Abstract] OR "manager*"[Title/Abstract] OR "boss"[Title/Abstract] OR "chief"[Title/Abstract] OR "superior"[Title/Abstract] OR "executive*"[Title/Abstract] OR "officer*"[Title/Abstract] OR "director*"[Title/Abstract] OR "leader behavio*"[Title/Abstract] OR "supervisor behavio*"[Title/Abstract] OR "leadership style*"[Title/Abstract]
#3	"digital*"[Title/Abstract] OR "virtual*"[Title/Abstract] OR "distant"[Title/Abstract]
#4	#2 AND #3
#5	"digital leadership"[MeSH Terms] OR "virtual leadership"[MeSH Terms] OR "distant leadership"[MeSH Terms] OR "eleadership"[Title/Abstract] OR "e-leadership"[Title/Abstract]
#6	#4 OR #5
#7	"mental health"[MeSH Terms] OR "occupational health"[MeSH Terms] OR "psychological health*"[Title/Abstract] OR "work related health*"[Title/Abstract] OR "psychosocial risk factor*"[Title/Abstract] OR "health outcome*"[Title/Abstract] OR "health indicator*"[Title/Abstract] OR "health behavio*"[Title/Abstract] OR "awareness"[Title/Abstract] OR "mindfulness"[Title/Abstract]
#8	"job satisfaction"[MeSH Terms] OR "work-life balance"[MeSH Terms] OR "motivation"[MeSH Terms] OR "self efficacy"[MeSH Terms] OR "work engagement"[MeSH Terms] OR "work performance"[MeSH Terms] OR "social support"[MeSH Terms] OR "well-being"[Title/Abstract] OR "wellbeing"[Title/Abstract] OR "work ability"[Title/Abstract] OR "employability"[Title/Abstract] OR "quality of work life"[Title/Abstract] OR "positive affectivity"[Title/Abstract] OR "positive feeling"[Title/Abstract] OR "organizational commitment"[Title/Abstract]
#9	"stress, psychological"[MeSH Terms] OR "occupational stress"[MeSH Terms] OR "psychological distress"[MeSH Terms] OR "burnout, professional"[MeSH Terms] OR "depressive disorder"[MeSH Terms] OR "anxiety"[MeSH Terms] OR "employee workload"[MeSH Terms] OR "fatigue"[MeSH Terms] OR "frustration"[MeSH Terms] OR "absenteeism"[MeSH Terms] OR "presenteeism"[MeSH Terms] OR "personnel turnover"[MeSH Terms] OR "sick leave"[MeSH Terms] OR "exhaustion"[Title/Abstract] OR "depression"[Title/Abstract] OR "burnout"[Title/Abstract] OR "stress reaction"[Title/Abstract] OR "strain*"[Title/Abstract] OR "mental strain*"[Title/Abstract] OR "stress*"[Title/Abstract] OR "work related stress"[Title/Abstract] OR "negative feeling"[Title/Abstract] OR "need for recovery"[Title/Abstract] OR "sadness"[Title/Abstract] OR "negative emotion*"[Title/Abstract] OR "psychological pressure"[Title/Abstract] OR "health impairment*"[Title/Abstract] OR "work load"[Title/Abstract] OR "monoton*"[Title/Abstract] OR "role conflict*"[Title/Abstract] OR "isolation"[Title/Abstract] OR "early retirement"[Title/Abstract] OR "disability pension"[Title/Abstract] OR "turnover"[Title/Abstract]
#10	#7 OR #8 OR #9
#11	#1 AND #6 AND #10
