# Supplementary material for: Virtual leadership in relation to employees' mental health, job satisfaction and perceptions of isolation: A scoping review
Source: Front Psychol. 2022 Oct 26;13:960955. doi: 10.3389/fpsyg.2022.960955 (PMC9644018; doi:10.3389/fpsyg.2022.960955)
Supplement: Supplementary Tables 3–6 — Critical appraisal results. [file Table_3.pdf]

Table 3. Critical appraisal of analytical cross-sectional studies (*n* = 13).

	Bentley et al. (2016)	Bregenzer & Jimenez (2021)	Golden (2006)	Golden & Veiga (2008)	Haines et al. (2002)	Karani & Mehta (2021)	Kelley & Kelloway (2012)	Kuruzovich et al. (2021)
1. Were the criteria for inclusion in the sample clearly defined?	✗	✓	✗	✗	✓	✓	✗	✓
2. Were the study subjects and the setting described in detail?	✓	✓	✓	✗	✓	✓	✓	✗
3. Was the exposure measured in a valid and reliable way?	✓	✓	(✓)	✓	✓	✓	✓	✓
4. Were objective, standard criteria used for measurement of the condition?	✓	(✓)	✓	✓	✗	✓	(✓)	(✓)
5. Were confounding factors identified?	(?)	(?)	✓	✓	✓	(?)	(?)	✓
6. Were strategies to deal with confounding factors stated?	N/A	N/A	✓	✗	✓	N/A	N/A	✓
7. Were the outcomes measured in a valid and reliable way?	✓	✓	✓	✓	✓	✓	✓	✓
8. Was appropriate statistical analysis used?	✓	✓	✓	✓	✓	✓	✓	✓

Table 3. *Cont.*

	Lurey & Raisinghani (2001)	Madlock (2012)	Mäkelä et al. (2019)	Nakrošienė et al. (2019)	Whitford & Moss (2009)
1. Were the criteria for inclusion in the sample clearly defined?	✗	✓	✗	✓	✗
2. Were the study subjects and the setting described in detail?	✓	✓	✓	✓	✓
3. Was the exposure measured in a valid and reliable way?	✓	✓	✓	(?)	✓
4. Were objective, standard criteria used for measurement of the condition?	✗	✓	(✓)	✗	✓
5. Were confounding factors identified?	(?)	(?)	✓	✓	✓
6. Were strategies to deal with confounding factors stated?	N/A	N/A	✓	✓	✓
7. Were the outcomes measured in a valid and reliable way?	✓	✓	✓	(?)	✓
8. Was appropriate statistical analysis used?	✗	✓	✓	✓	✓

Note. ✓ = Yes, ✗ = No, (✓) = Partly, (?) = Unclear, N/A = Not applicable.

Table 4. Critical appraisal of qualitative studies (*n* = 4).

	Kirkman et al. (2002)	Kurland & Cooper (2002)	Poulsen & Ipsen (2017)	Ruiller et al. (2019)
1. Is there congruity between the stated philosophical perspective and the research methodology?	(?)	✓	(?)	(?)
2. Is there congruity between the research methodology and the research question or objectives?	✓	✓	✓	✓
3. Is there congruity between the research methodology and the methods used to collect data?	✓	✓	✓	✓
4. Is there congruity between the research methodology and the representation and analysis of data?	✓	✓	✓	✓
5. Is there congruity between the research methodology and the interpretation of results?	✓	✓	✓	✓
6. Is there a statement locating the researcher culturally or theoretically?	✓	✗	✓	✗
7. Is the influence of the researcher on the research, and vice- versa, addressed?	✗	✗	✗	✗
8. Are participants, and their voices, adequately represented?	✓	✓	✓	✓

9. Is the research ethical according to current criteria or, for recent studies, and is there evidence of ethical approval by an appropriate body?	✗	✗	✗	✗
10. Do the conclusions drawn in the research report flow from the analysis, or interpretation, of the data?	✓	✓	✓	✓

Note. ✓ = Yes, ✗ = No, (✓) = Partly, (?) = Unclear, N/A = Not applicable.

Table 5. Critical appraisal of cohort studies (*n* = 1).

	De Vries et al. (2019)
1. Were the two groups similar and recruited from the same population?	✓
2. Were the exposures measured similarly to assign people to both exposed and unexposed groups?	✓
3. Was the exposure measured in a valid and reliable way?	(?)
4. Were confounding factors identified?	✗
5. Were strategies to deal with confounding factors stated?	N/A
6. Were the groups/ participants free of the outcome at the start of the study (or at the moment of exposure)?	✓
7. Were the outcomes measured in a valid and reliable way?	✓
8. Was the follow up time reported and sufficient to be long enough for outcomes to occur?	✓
9. Was follow up complete, and if not, were the reasons	✓

to loss to follow up described and explored?	
10. Were strategies to address incomplete follow up utilized?	✗
11. Was appropriate statistical analysis used?	✓

Note. ✓ = Yes, ✗ = No, (✓) = Partly, (?) = Unclear, N/A = Not applicable.

Table 6. Critical appraisal of quasi-experimental studies (*n* = 1).

	Konradt et al. (2003)
1. Is it clear in the study what is the ‘cause’ and what is the ‘effect’ (i.e. there is no confusion about which variable comes first)?	✓
2. Were the participants included in any comparisons similar?	✓
3. Were the participants included in any comparisons receiving similar treatment/care, other than the exposure or intervention of interest?	✓
4. Was there a control group?	✓
5. Were there multiple measurements of the outcome both pre and post the intervention/exposure?	✗
6. Was follow up complete and if not, were differences between groups in terms of their follow up adequately described and analyzed?	N/A
7. Were the outcomes of participants included in any comparisons measured in the same way?	✓

8. Were outcomes measured in a reliable way?	✓
9. Was appropriate statistical analysis used?	✓

Note. ✓ = Yes, ✗ = No, (✓) = Partly, (?) = Unclear, N/A = Not applicable.
